# α-Amylase-Mediated Antibiotic Degradation and Sequestration in *Pseudomonas aeruginosa* Biofilm Therapy

**DOI:** 10.3390/antibiotics14090941

**Published:** 2025-09-18

**Authors:** Robert K. Murray, Allison E. Martin, Sarah Zipkowitz, Nusrat Jahan, Tony D. Davis, Whitni K. Redman

**Affiliations:** 1 Department of Biological Sciences, Binghamton University, Binghamton, NY 13902, USA; rmurra10@binghamton.edu (R.K.M.); zipkowitzsarah@gmail.com (S.Z.); njahan22@binghamton.edu (N.J.); 2 First-Year Research Immersion Program, Binghamton University, Binghamton, NY 13902, USA; 3 Department of Pharmaceutical Sciences, School of Pharmacy and Pharmaceutical Sciences, Binghamton University, Binghamton, NY 13902, USA; amart225@binghamton.edu (A.E.M.); tddavis@binghamton.edu (T.D.D.)

**Keywords:** glycoside hydrolase, biofilm, treatment, antibiotic, *Pseudomonas aeruginosa*

## Abstract

Background: As of 2022, 80% of all documented microbial infections are biofilm-associated: communities of microorganisms adhered to a surface and enclosed in a complex extracellular polymeric substance (EPS). The EPS acts as a physical barrier protecting the bacteria from antimicrobial agents and host immune responses. To combat this hurdle, the application of glycoside hydrolases (GH) has been investigated due to their ability to cleave particular structural polysaccharides within the EPS, thus breaking down the protective barrier and improving antibiotic clearance. While various studies demonstrate the capacity of GHs to improve antibiotic efficacy against biofilms in combination, there is clear differential success between these treatments depending on the GH and antibiotic chosen. Due to the overlap of GH targets and antibiotic structures, it is imperative to ensure that the antibiotics in combinatorial treatments are not degraded by the GH. Methods: This study aimed to screen the GH α-amylase produced from *Aspergillus oryzae* (AO) and *Bacillus subtilis* (BS), combined with various antibiotics from different classes, charges, and mode of actions by determining MICs. against the bacterium *Pseudomonas aeruginosa* (PA) of 6 antibiotics with or without α-amylase and treat 2-day PA biofilms with antibiotics with or without GHs. Liquid Chromatography Tandem Mass Spectrometry (LC-MS/MS) stability assays and Differential Scanning Fluorimetry (DSF) were conducted to determine antibiotic and GH degradation as well as antibiotic sequestration. Results: Increased MICs in the presence of GHs as well as decreased antibiotic clearance against 2-day biofilms were suggestive of antibiotic degradation. LC-MS/MS stability assays of tetracycline and ciprofloxacin in the presence and absence of α-amylase further demonstrated the α-amylase-mediated antibiotic sequestration. Differential scanning fluorimetry (DSF) assays confirmed α-amylase-antibiotic interactions. Conclusions: This study suggests that α-amylase is capable of degrading and sequestering a variety of antibiotics, and the degree to which these phenomena occur varies depending upon the source of the GH. As a potential treatment for biofilm-associated infections, it is imperative that the GH + antibiotic combinations are determined compatible prior to clinical use.

## 1. Introduction

One of the most significant problems in healthcare is the rapid emergence of antimicrobial-resistant infections. In 2019 alone, 1.27 million deaths were associated with antimicrobial resistance (AMR) worldwide, making it a leading cause of death around the world [1]. Many factors contribute to the spread of AMR, including intrinsic factors such as bacteria’s generally rapid mutation rate and extrinsic factors such as the overuse of antibiotics in the clinic and in agriculture [2,3,4,5]. Among these factors, the ability of these infections to form biofilms has been the subject of extensive research due to the additional challenges in treatment they pose [6]. Biofilms are mono- or polymicrobial communities that adopt a largely sedentary phenotype attached to a biotic or abiotic substrate and are enclosed in an extracellular polymeric substance (EPS). Biofilm infections possess various qualities that further compound the struggle of AMR in healthcare. For example, the EPS matrix can serve as a physical barrier protecting the bacteria from host immune responses and antimicrobial agents, directly reducing the effectiveness of antibiotic treatment [7,8]. Additionally, the sedentary nature of the biofilm results in a slowing of the metabolic activity of the bacteria, further lowering the efficacy of antimicrobial treatments that target specific critical mechanisms [9].

Due to the advantages of adopting a biofilm life cycle, nearly 80% of all documented bacterial infections are associated with biofilms [10,11]. In clinical settings, biofilms are responsible for many infections of medical devices such as breast implants, joint prostheses, and ventricular assist devices [12]. Non-device biofilm infections are common in many disease states such as diabetic foot ulcers or other chronic wounds, urinary tract infections, and periodontitis [13,14,15]. The variety of biofilm-forming human pathogens is vast, including bacteria such as *Escherichia coli*, *Vibrio* sp., *Staphylococcus aureus*, and *Staphylococcus epidermidis* [16,17,18]. Among these pathogens, the Gram-negative bacterium *Pseudomonas aeruginosa* is one of the most prevalent and is commonly associated with various disease states. It is notorious for the chronic infection of cystic fibrosis (CF) patients, triggering a pronounced, prolonged host immune response that causes inflammatory damage to the lungs [19]. Although some anti-biofilm therapeutics have found success in the clinic [20,21,22,23], much research on the biofilm lifestyle is needed to develop novel ways to disrupt its growth and spread in the host.

Analysis of the general biofilm life cycle provides useful information that can be used to develop anti-biofilm treatments. A biofilm develops in five distinct stages: reversible attachment, irreversible attachment, maturation I and II, and dispersion [24]. Researchers have taken particular interest in dispersion, the concluding stage of biofilm development, as it is hypothesized that the dispersed bacteria are more susceptible to treatment [25,26,27,28]. Thus, biofilm dispersion has been a promising direction for anti-biofilm treatment development. There are two defined modes of dispersion—active and passive—each exhibiting different implications for treatment effectiveness.

Active dispersion involves the release of triggers by biofilm bacteria, leading to the expression of enzymes that break down the EPS, resulting from internal or environmental cues [25,27]. A prime example of an intrinsic active dispersion cue is *cis*-2-decenoic acid (*cis*-DA), which has been extensively researched for its direct involvement in the departure from the biofilm phenotype, primarily in *P. aeruginosa*. An increased concentration of *cis*-DA decreases intracellular cyclic-di-GMP levels, causing dispersion [29,30,31]. Environmental cues for active dispersion include toxins such as nitric oxide and nutrient availability cues such as glutamine and pyruvate depletion, and exogenous exposure of these cues to biofilms has been shown to improve infection clearance when combined with antibiotics [32,33,34,35]. However, actively dispersed cells are suggested to express a more virulent “third phenotype,” possessing characteristics of biofilm and planktonic cells [24,36]. Although it is not entirely known whether these cells are more tolerant to antimicrobial treatment, their more virulent properties are a cause of concern regarding the direction of inducing active dispersion for effective anti-biofilm treatments [24,37,38].

Passive dispersion involves external disturbances that physically or enzymatically breaks down the EPS or otherwise detach the biofilm [25,27]. Physical debridement of biofilm infections is commonly practiced in the clinic, especially for those suffering from biofilm-associated chronic wounds [39]. Although these methods have seen success in the removal of biofilms from a wound, they are often expensive, painful for patients, and could result in collateral damage to healthy tissue like blood vessels, nerves, and tendons [40]. Enzymatic passive dispersion involves using specific enzymes to break down critical structural components of the EPS matrix and directly disperse the biofilm [41]. Passive dispersion is the favored mode of biofilm dispersion to be induced in clinical settings, as passively dispersed cells are hypothesized to not be triggered by intrinsic biochemical means to exhibit the “third phenotype” seen in actively dispersed cells. This would make passively dispersed cells less virulent and potentially less tolerant to antibiotics [28]. Additionally, agents of active dispersion such as nitric oxide can be toxic to host organisms in elevated concentrations, which can result in fatal conditions like pulmonary edema and cardiac arrest [25]. Conversely, enzymatic passive dispersal agents are suggested to be safe for use, having no negative effect on wound healing rates or cell toxicity [28]. Examples of enzymatic anti-biofilm drugs used in the clinic include Pulmozyme, a DNAse targeting extracellular DNA within the EPS of *P. aeruginosa* biofilms in CF patients [20], and DispersinB, a glycoside hydrolase targeting the dPNAG exopolysaccharide located in the EPS of a variety of biofilm species [21,23].

Among the variety of enzymatic passive dispersion agents researched, the glycoside hydrolase (GH) class of enzymes has been the subject of research as a promising component of an enzyme + antibiotic combinatorial treatment [42,43,44,45]. One GH that is researched for its anti-biofilm capability is α-amylase, which cleaves α-1,4-glycosidic linkages in polysaccharide structures such as starch [46]. Its catalytic mechanism has made it a highly marketable enzyme with uses in baking, brewing, and cleaning, and has more recently been studied for its potential application in antimicrobial medicine [46,47]. α-1,4-Glycosidic linkages are also found in some structural polysaccharides within EPS matrices, including the Pel polysaccharide of *P. aeruginosa* biofilms [48,49], making α-amylase a promising therapeutic anti-biofilm agent when combined with antibiotics. An improvement in antibiotic clearance of *P. aeruginosa* by simultaneous treatment with α-amylase has already been documented in vivo and in vitro [42,49,50,51,52], but further optimization of combinatorial α-amylase + antibiotic treatment is critical before safe use in clinical settings.

In the process of optimizing GH + antibiotic treatments against microbial biofilms, concerns that must be considered include those that result in a loss of antibiotic efficacy. In these cases, the passively dispersed bacteria will not be killed by the antibiotic and re-circulate in the host. This may lead to an overwhelming burden of planktonic bacteria in the host, resulting in sepsis and the potential re-establishment of biofilms throughout the body [27]. The incompatibility of GHs and antibiotics has been suggested previously [43,53]. One such criterion that should be considered is the degradation of antibiotic structures by the GH. This may occur in cases where the GH’s target linkage is also present in the antibiotic structure. When combined with antibiotics in an anti-biofilm treatment, the GH may target not only the specific EPS polysaccharide but also the antibiotic possessing the same linkage in its structure. Presently, there is a lack of anti-biofilm therapeutic research investigating this phenomenon, and GH-mediated antibiotic degradation has yet to be properly documented and confirmed.

This study aims to investigate the phenomenon of antibiotic degradation by GHs due to structural overlap between biofilm EPS polysaccharides and antibiotics. We selected six antibiotics to test for GH degradation based on variations in microbial effect, charge, class, and target mechanism or structure. α-Amylase was selected as the GH to be studied due to its ability to improve antibiotic clearance of *P. aeruginosa* biofilms in vitro and in vivo [42,43,49]. For this reason, *P. aeruginosa* was chosen as our biofilm-forming model organism.

α-Amylase from the fungus *Aspergillus oryzae* (AO) and bacterium *Bacillus subtilis* (BS) were tested to determine whether different GH analogs exhibited differential degradative ability. A fungal and bacterial homolog were selected for this study because they differ in their amino acid sequence and therefore potentially differ in their folded protein structure which can consequently result in differences in biochemical properties [54]. For example, the optimal pH ranges for α-amylase AO and BS are 5–7 and 6–10, respectively [54,55], and the optimal temperature ranges are 25–35 °C and 37–50 °C, respectively [55]. These differences in optimal system conditions could result in varying antibiotic interaction.

Minimum inhibitory concentrations (MIC) were determined for antibiotics only as a baseline, then for the antibiotic + α-amylase combinations to observe changes in the MICs upon combinatorial treatment with each amylase analog. The six antibiotics were analyzed via liquid chromatography–tandem mass spectrometry (LC-MS/MS) with and without α-amylase homologs to understand the changes in MIC observed in the presence of α-amylase. LC-MS/MS stability assays of antibiotics were conducted with and without the presence of α-amylase AO and BS for comparative analysis. This method aimed to quantitatively confirm the degradation of antibiotic structures by α-amylase, as well as the degree of degradation of each antibiotic with α-amylase AO and BS. Differential scanning fluorimetry (DSF) assays were also used to confirm antibiotic-amylase interactions. Lastly, we aimed to confirm this phenomenon with in vitro methodology via cell viability quantification using a 24-well plate 2-day biofilm model with a 24 h treatment time. With this study, we hope to demonstrate the importance of optimizing GH + antibiotic combinatorial treatments against microbial biofilm infections due to GH-mediated antibiotic degradation. Additionally, we stress the importance of antibiotic degradation as one of the many variables to consider when determining optimal GH + antibiotic combinations before clinical use.

## 2. Results

### 2.1. α-Amylase AO and BS Did Not Improve Antibiotic Clearance

To determine if α-amylase could improve antibiotic clearance, *P. aeruginosa* biofilms were treated with a media control (LB), antibiotic alone, or antibiotic with either 500 U/mL α-amylase AO or 10% α-amylase BS. The antibiotics gentamicin at a sub-inhibitory concentration (0.78 µg/mL) and levofloxacin at the MIC concentration (1.5 µg/mL) were used. A sub-inhibitory and inhibitory concentration was used to determine if α-amylase could improve killing at a lower antibiotic concentration. Then, 2-day PAO1 biofilms were established then treated with their respective treatment for either 2 or 24 h. The remaining biofilm was washed with saline, collected, and viable cells were quantified as CFU/mL. Comparisons were made against an LB treatment control group and antibiotic alone treatment control group.

**Figure 1:**
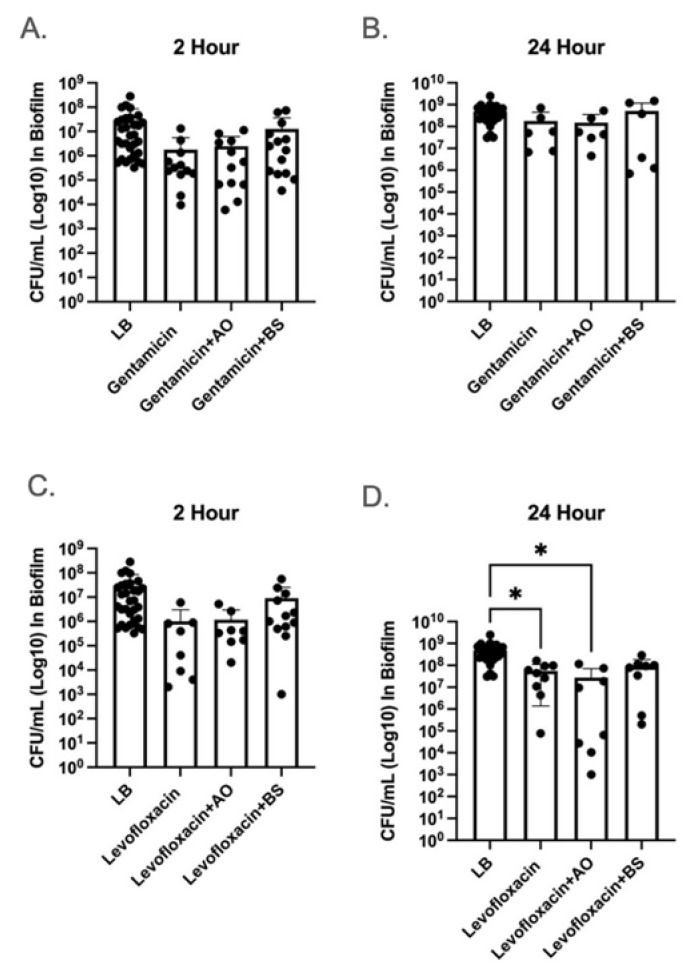
The 2 and 24 h combinatorial treatments of α-amylase and antibiotic did not improve antibiotic clearance of biofilm. The efficacy of the combinatorial α-amylase and antibiotic treatment was determined utilizing a 2 (**A**,**C**) and 24 (**B**,**D**) hour treatment of 48 h PAO1 biofilms established in 24-well plates. Biofilms were treated with LB (media control), 0.78 µg/mL gentamicin (**A**,**B**) or 1.5 µg/mL levofloxacin (**C**,**D**) with and without α-amylase AO or α-amylase BS. Following treatment, biofilm samples were washed, serially diluted, and drop-plated to enumerate CFU/mL. Statistical significance was determined via one-way ANOVA and Tukey–Kramer multiple-comparison test. * *p* < 0.05. *n* = 6–24.

Although not significant, gentamicin (2 h 1.7 × 10^6^ CFU/mL, 24 h 1.8 × 10^8^ CFU/mL) alone did exhibit a decrease in bacterial load compared to LB alone (2 h 2.8 × 10^7^ CFU/mL, 24 h 4.9 × 10^8^ CFU/mL) for both 2 h (*p* = 0.192) and 24 h (*p* = 0.503) treatments. Gentamicin combined with α-amylase AO or BS did not significantly reduce the remaining biofilm CFU/mL after either 2 or 24 h treatment compared to LB control, nor the gentamicin-only treatment group (*p* > 0.05) (Figure 1A,B). The addition of either α-amylase AO and BS for a 2 h treatment actually increased the bacterial load remaining in the biofilm compared to gentamicin alone (1.6 × 10^6^ CFU/mL) with 2.4 × 10^6^ CFU/mL and 1.2 × 10^7^ CFU/mL, respectively. α-amylase BS exhibited a similar trend at 24 h treatments with a bacterial load of 5.1 × 10^8^ CFU/mL compared to gentamicin alone (1.8 × 10^8^ CFU/mL). Although not significant, the addition of α-amylase AO did decrease the remaining bacterial load compared to gentamicin alone, with 1.5 × 10^8^ CFU/mL remaining.

Levofloxacin alone did not significantly reduce bacterial load compared to the LB control at 2 h (*p* = 0.346) but did show significant reduction after 24 h treatments (*p* = 0.021) (Figure 1C,D). Although not significant, the combinatorial treatments at 2 h again exhibited an increase in bacterial burden remaining in the biofilm after the 2 h treatment for both α-amylase AO (1.17 × 10^6^ CFU/mL) and BS (9.01 × 10^6^ CFU/mL) compared to levofloxacin (1.0 × 10^6^ CFU/mL) alone. At 24 h, levofloxacin + α-amylase AO exhibited significant reduction compared to the LB control (*p* = 0.019) but not to the antibiotic alone (*p* = 0.998), suggesting that α-amylase AO did not improve antibiotic clearance. Similarly to gentamicin, α-amylase AO (2.7 × 10^7^ CFU/mL) reduced the bacterial burden remaining in the biofilm compared the antibiotic alone (5.6 × 10^7^ CFU/mL) while α-amylase BS exhibited an increase in remaining bacteria (8.88 × 10^7^ CFU/mL).

The increase in bacterial load remaining in the biofilm in the combinatorial treatment compared to the antibiotic alone could be because the enzymatic treatment of samples has been shown to increase bacterial counts, the potential of enzyme/antibiotic interactions inhibiting the antimicrobial activity, or EPS sequestering the antibiotics.

### 2.2. Exposure to α-Amylase AO and BS Resulted in Altered MIC Values

To assess the effects of α-amylase on antibiotic efficacy, MICs were performed with either 500 U/mL α-amylase AO or 10% α-amylase BS added to the antibiotic treatment. MICs were conducted for 24 h as the standard MIC incubation time and covers the 30 min exposure previously used in in vivo studies [26,28] as well as 2 h exposure used in previous in vitro studies [42,49] and the 24 h exposure utilized in this study. Comparison of the combinatorial treatment MICs to those with only antibiotics allowed for a basic evaluation of the synergistic and inhibitory effects of α-amylase on antibiotic activity (Table 1). Increases in MIC with α-amylase suggest inhibitory effects, and decreases suggest synergistic effects.

Increases in MICs were observed for three antibiotics in the presence of both α-amylase homologs: ciprofloxacin increased from an MIC of 0.4 µg/mL to 0.78 µg/mL with α-amylase AO and 1.563 µg/mL with α-amylase BS; levofloxacin increased from 1.5 µg/mL to 3.125 µg/mL with α-amylase AO and 6.25 µg/mL with α-amylase BS; tetracycline increased from 31.25 µg/mL to >1000 µg/mL with α-amylase AO and 500 µg/mL with α-amylase BS (Table 1, Figure S1). This suggests that the presence of α-amylase, regardless of the enzyme source, in these antibiotic treatments yields inhibitory effects on the overall efficacy of the antibiotic. These findings support the hypothesized phenomenon of α-amylase-mediated antibiotic degradation that resulted in increased MICs. Hypothetically, α-Amylase-mediated degradation involves the antibiotic possessing structural elements that are similar to the enzyme’s target, resulting in the antibiotic binding to active or allosteric sites, breakdown of the compound’s structure, and a subsequent dampening or loss of function.

An increased MIC also occurred for two antibiotics in the presence of just one α-amylase homolog: tobramycin increased from 6.25 µg/mL to 25 µg/mL with α-amylase BS, while α-amylase AO did not affect the MIC; gentamicin increased from 3.125 µg/mL to 25 µg/mL with α-amylase BS, while α-amylase AO did not affect the MIC (Table 1). A possible explanation for this may be that the α-amylase BS structure results in a greater affinity for the two antibiotics than α-amylase AO. Additionally, a decrease in colistin sulfate MIC is observed in the presence of α-amylase BS, from 31.25 µg/mL to 15.63 µg/mL (Table 1). In this case, α-amylase BS may have a synergistic effect when combined with colistin sulfate, resulting in an enhanced clearance of the bacteria.

### 2.3. Sequestration and Degradation of Antibiotics by α-Amylase AO and BS

The increased MICs of some of the combinatorial antibiotic + α-amylase treatments compared to antibiotic alone raise the possibility that antibiotics may be unstable in the presence of amylase. To investigate this, we conducted quantitative liquid chromatography–tandem mass spectrometry (LC–MS/MS) analyses of antibiotics 1 µM (levofloxacin, ciprofloxacin, and tetracycline), 5 µM (colistin) or 7 µM (tobramycin and gentamicin) treated with 1% α-amylase for 22 h at 37 °C. Antibiotics without amylase were included as a comparison group.

**Figure 2:**
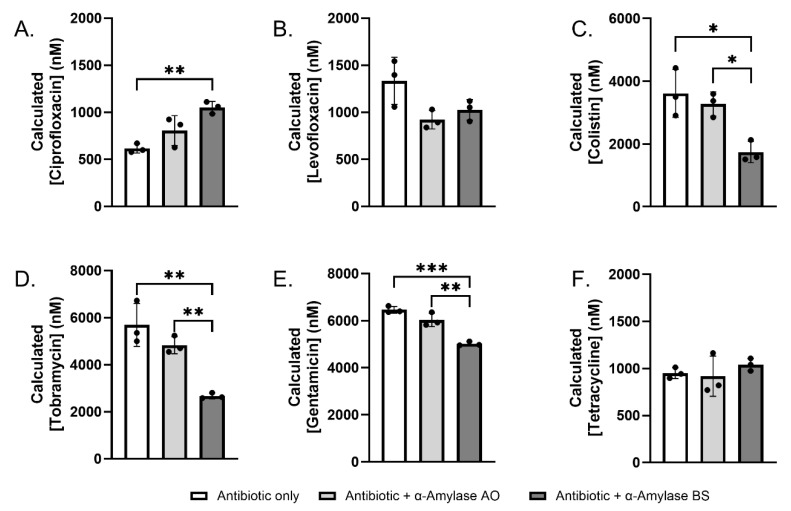
Amylase-mediated sequestration or degradation of antibiotics. Stability of (**A**) 1 µM ciprofloxacin, (**B**) 1 µM levofloxacin, (**C**) 5 µM colistin, (**D**) 7 µM tobramycin, (**E**) 7 µM gentamicin, and (**F**) 1 µM tetracycline in the absence or presence of 1% α-amylase AO or 1% α-amylase BS for 22 h at 37 °C in 0.85% NaCl. Following incubation, protein was precipitated, and the supernatant was lyophilized and resuspended in deionized water prior to LC-MS/MS analysis. Statistical significance was determined via one-way ANOVA and Tukey’s multiple-comparison test. * *p* < 0.05; ** *p* < 0.01; *** *p* < 0.001, *n* = 3.

In the absence of amylase, ciprofloxacin levels decreased to 615.3 ± 49.0 nM (Figure 2A). This is consistent with published reports of precipitation of ciprofloxacin in normal saline at 37 °C [56]. Interestingly, co-treatment of α-amylase BS significantly increased ciprofloxacin (1051.2 ± 64.0 nM, *p* = 0.0049) levels. α-Amylase AO also increased ciprofloxacin levels (805.7 ± 158.6 nM, *p* = 0.1370), though not significantly. This suggests that α-amylase BS may sequester ciprofloxacin, thereby protecting it from degradation. We also tested levofloxacin and tetracycline, which remained stable under the assay conditions and were unaffected by either amylase (Figure 2B,F). Colistin, on the other hand, exhibited a decrease in concentration in the presence of BS (1740.6 ± 338.1 nM, *p* = 0.0118), suggestive of antibiotic degradation occurring (Figure 2C). Similarly to colistin, this trend was also observed for tobramycin (2670.2 ± 119.5 nM, *p* = 0.0016) and gentamicin (5014.2 ± 90.0 nM, *p* = 0.0002) (Figure 2D,E).

### 2.4. Antibiotics Interact with α-Amylase AO and BS

To determine whether antibiotics bind to amylase, we conducted differential scanning fluorimetry (DSF), a biophysical technique to evaluate the stability of protein–ligand and protein–protein complexes [57,58]. DSF is a widely used technique to identify ligands that bind to purified proteins [59,60,61].

We first conducted DSF assays with each α-amylase in various buffers to determine if they were amenable to the assay. α-amylase AO displayed T_m_ 59.8 ± 0.5 °C and 58.6 ± 0.5 °C in normal saline and PBS, respectively (Figure S2). In 50 mM HEPES (pH 7.4) we observed a significant increase in α-amylase AO T_m_ of 62.9 ± 0.4 °C. By comparison, α-amylase BS displayed higher melting temperatures of 62.8 ± 0.6 °C, 70.9 ± 0.4 °C, and 71.9 ± 0.4 °C in normal saline, PBS, and HEPES, respectively.

**Figure 3:**
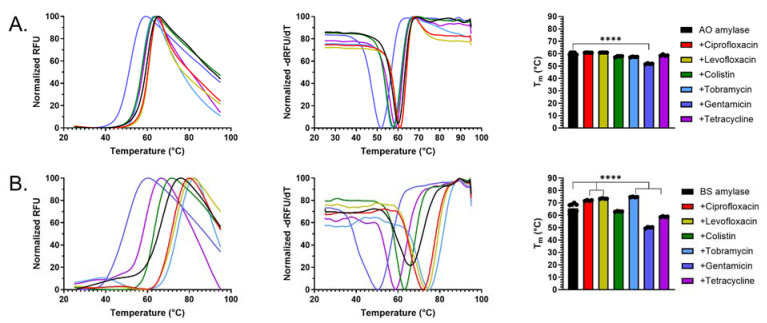
Differential scanning fluorimetry reveals that antibiotics bind amylases. Normalized fluorescence of SYPRO Orange (left), first-derivative plots (center), and melting temperatures (right) for antibiotics (1 mM) in the presence of (**A**) 1.0 mg/mL α-amylase AO or (**B**) 1.0 mg/mL α-amylase BS in 0.85% NaCl. Statistical significance was determined via one-way ANOVA and Tukey multiple-comparison test. ****, *p* < 0.0001, *n* = 4.

Given the robust thermal melt curves, we next conducted DSF assays with each amylase in the presence of ciprofloxacin, levofloxacin, colistin sulfate, tobramycin, gentamicin, or tetracycline to assess binding. The addition of gentamicin to α-amylase AO significantly decreased protein stability compared to α-amylase AO alone (ΔT_m_ ~ 8 °C) (Figure 3A), suggesting that this aminoglycoside binds the amylase. Interestingly, there were no significant differences in T_m_ when α-amylase AO was treated with tobramycin or any of the other antibiotics. Gentamicin and tetracycline also destabilized α-amylase BS (ΔT_m_ 7–8 °C), while tobramycin and both fluoroquinolones significantly increased α-amylase BS stability (ΔT_m_ 6–8 °C), again suggestive of antibiotic-α-amylase interactions (Figure 3B).

## 3. Discussion

Among the factors contributing to the prevalence of AMR, the ability of bacterial pathogens to form microbial biofilms is one of the most significant and biologically challenging to address. Biofilm infections can be 10–1000 times more resistant to traditional antimicrobial treatment than non-biofilm infections [62], with much resistance attributed to the characteristic EPS. The GH α-amylase hydrolyzes α-1,4 glycosidic linkages such as the Pel polysaccharide found in *Pseudomonas aeruginosa* biofilms, making it a promising anti-biofilm candidate via exogenous EPS breakdown [48,49]. Although α-amylase has been shown to trigger biofilm dispersion in vitro and in vivo, allowing greater antimicrobial efficacy [28,42,43,49], some combinations of α-amylase and antibiotics exhibit sub-optimal success. In the process of optimizing GH + antibiotic combinatorial treatments, it is important to consider the possibility of interactions between α-amylase and antibiotics through which the overall treatment efficacy is lowered. The goal of this study was to document and define the presence and abundance of inhibitory α-amylase + antibiotic interactions when used as combinatorial treatments against *P. aeruginosa* biofilms.

In vitro cell viability assays revealed that the addition of α-amylase AO or BS did not significantly improve the efficacy of levofloxacin or gentamicin against PAO1 biofilms after 2 and 24 h treatment periods compared to antibiotic-only and LB control groups (Figure 1). Several factors may explain the lack of antibiotic activity improvement upon the addition of α-amylase, such as our proposed inhibitory interactions between the enzyme and antibiotic, the increased antibiotic tolerance of passively released biofilm cells [9,63,64], and the inflation of bacterial counts via enzymatic treatment [65].

MIC assays revealed that the degree of MIC increase varied depending on the antibiotic, as well as the homolog of α-amylase used. This phenomenon is supported by the results for tobramycin and gentamicin, in which combination with α-amylase AO did not yield MIC increases, whereas combination with α-amylase BS increased MICs by factors of 4 and 8, respectively (Table 1). In this instance, it is inferred that α-amylase BS has a greater affinity for these two antibiotics than α-amylase AO, resulting in greater degradation. Also, colistin sulfate MIC decreased in the presence of α-amylase BS, which is the only instance of an MIC decrease due to α-amylase combination. Consequently, the combination of colistin sulfate and 10% α-amylase BS may be a promising candidate for effective *P. aeruginosa* biofilm infection treatment, and further work should assess its efficacy in vivo.

LC-MS/MS stability assays of the six antibiotics with or without α-amylase revealed that α-amylases may sequester or degrade the antibiotic (Figure 2). Similarly to previous findings, we also showed that the degree of degradation and sequestration of a particular antibiotic depends on the homolog of α-amylase used, as α-amylase BS significantly increased or decreased the concentration of recovered antibiotic after incubation compared to the antibiotic alone or with α-amylase AO. The previous literature suggests that antibiotic structures possessing multiple hydrogen bond donors may be more susceptible to inhibitory interactions with α-amylase [53,66], whether they impact α-amylase activity, antibiotic activity, or a combination of both. Regardless, it can be inferred that certain antibiotics may be degraded or sequestered by α-amylase at a greater rate than others as a result of their structure. Since gentamicin and tobramycin contain glycosidic linkages, the degradation by α-amylases is noteworthy, and suggests these hydrolases may recognize additional cleavage sites beyond canonical α-1,4 glycosidic linkages. Secondly, the organismal source of α-amylase must be considered, as this directly affects the degree to which antibiotic or α-amylase activity is inhibited.

The DSF assays further support the proposed ideas that α-amylase differentially interacts with antibiotics, and that the degree to which these interactions occur is partially dependent on the homolog of α-amylase used. Overall, the results show that α-amylase AO may be a better homolog choice for α-amylase + antibiotic treatment of PA biofilms, as antibiotic levels were not significantly altered in the presence of AO. In contrast, treatment of five of the six antibiotics with α-amylase BS significantly altered antibiotic levels. Additionally, the results suggest that α-amylase may synergize poorly with gentamicin in a combinatorial α-amylase + antibiotic treatment, as this combination resulted in a significant decrease in stability of both α-amylase AO and BS.

## 4. Materials and Methods

### 4.1. Bacterial Strains and Culture Preparation

Bacterial experiments were performed using the *Pseudomonas aeruginosa* PAO1 strain [28,49,65]. PAO1 overnight cultures were prepared from a frozen stock in 20 mL full strength Lennox Broth (LB) growth medium (Sigma Aldrich^®^ St. Louis, MO, USA; CAT #45-L3022) in 250 mL Erlenmeyer flasks and incubated at 37 °C and 220 rpm shaking for 16–18 h.

### 4.2. α-Amylase Preparation

α-amylase sourced from the fungus *Aspergillus oryzae* (Sigma-Aldrich^®^; CAT #A9857) (AO) and the bacterium *Bacillus subtilis* (MP Biomedicals^®^ Solon, OH, USA; CAT #9000-90-2) (BS) were utilized in this study. α-Amylase AO was prepared in 500 U/mL solutions and α-amylase BS was prepared in 10% *w/v* solutions as used previously [28,49,65]. Full strength LB was used to suspend the solutions and served as the vehicle control. α-amylase solutions were incubated immediately before use at 37 °C for 30 min to allow for enzyme activation. The same procedure was followed for α-amylase + antibiotic combinatorial treatment solutions. Heat-inactivated (HI) α-amylase was prepared using the standard concentrations in 15 mL Falcon tubes and boiled in a water bath for 10 min.

### 4.3. Antibiotic Preparation

Six antibiotics were selected based on microbial effect, charge, class, and target mechanism or cellular structure (Table S1). Then, 10,000 μg/mL antibiotic stock solutions, with the exception of tetracycline, were prepared by dissolving the antibiotic in deionized water, followed by filter sterilization using a 0.22 μm nanopore syringe filter (M2Sci Allendale, MI, USA; CAT #371-2215-OEM). Next, 10,000 μg/mL tetracycline stock solutions were suspended in 100% ethanol, followed by filter sterilization using a 0.22 μm nanopore syringe filter, then covered with aluminum foil to protect from light. All antibiotic stock solutions were stored at −20 °C. Antibiotics were prepared for bacterial experiments by diluting the stock solution in full strength LB immediately before treatment. α-amylase + antibiotic combinatorial treatment solutions were prepared similarly, then incubated for 30 min at 37 °C and 220 rpm shaking. Then, 24 h α-amylase + antibiotic pre-treatments were incubated 24 h before biofilm treatment.

### 4.4. Forty-Eight-Hour Well-Plate Biofilm Growth

In vitro cell viability experiments utilized a 24-well plate biofilm growth model described in the previous literature [27,28,49]. An overnight was used to create a 1% subculture in full strength LB, which was then incubated for 2.5 h at 37 °C and 220 rpm shaking. The subculture was adjusted to OD_600_ 0.4, followed by a 1:100 dilution in full strength LB to generate a 10^6^ CFU/mL inoculant. Wells were inoculated with 800 μL of the 10^6^ CFU/mL inoculant. Inoculated plates were incubated for 48 h at 37 °C and 80 rpm shaking. To determine the efficacy of the combinatorial treatments, 48 h PAO1 biofilms were treated. Treatments were prepared as previously described, with antibiotic concentrations reflecting their determined MICs. Treatment classes included: untreated; antibiotic; α-amylase; antibiotic + α-amylase combinatorial treatments; and antibiotic + α-amylase combinatorial treatments (24 h incubation). Biofilm treatment involved careful removal of the unattached cells, a rinse with 0.85% saline to remove additional non-biofilm cells, followed by 1 mL treatment. After the treatment was added, plates were incubated at 37 °C and 80 rpm shaking for 2 or 24 h. After incubation, the supernatant was collected, followed by two 1 mL 0.85% saline rinses of the attached biofilm to remove the remaining treatment. After completing the washes, 1 mL of 0.85% saline was added to each well and the plate was water-sonicated for 30 min on the high setting (Fisher Scientific Waltham, MA, USA; UltraSonic Bath 2.8 L) to debride the cells remaining in the biofilm. After collecting the biofilm samples, the supernatant and biofilm samples were serially diluted in 0.85% saline, and then drop-plated on full strength LB 1.5% agar plates. Drop plates were incubated at 37 °C for 24 h. After incubation, supernatant and biofilm CFU/mL were quantified.

### 4.5. Minimum Inhibitory Concentration (MIC)

Minimum inhibitory concentration determinations were made for all six antibiotics using 96-well plate broth dilution. Then, 10,000 μg/mL antibiotic stocks were diluted to 1000 or 100 μg/mL in LB; these concentrations were used to perform 1:2 serial dilutions down the plate in LB. The resulting concentration ranges were 1000–2 μg/mL and 100–0.2 μg/mL, respectively. The wells were then inoculated with 20 μL of PAO1 overnight culture adjusted to OD_600_ 0.5. A column of LB + inoculant with no antibiotics served as the positive control, with a column of just LB as the negative control. Inoculated plates were incubated at 37 °C and 220 rpm shaking for 24 h. After incubation, a plate-reading spectrophotometer (SpectraMax^®^ San Jose, CA, USA; Molecular Devices) recorded absorbance at 600 nm wavelength to measure cell growth. MICs were defined as the lowest concentration in which the absorbance was similar to that of the LB-only negative control [67].

### 4.6. α-Amylase Mediated Antibiotic Interactions

MICs were repeated in the presence of α-amylase. The antibiotic solutions were combined with either homolog of α-amylase to make a combinatorial treatment. Then, 10,000 μg/mL antibiotic stock solutions were first diluted to 1000 or 100 μg/mL working concentrations in full strength LB. α-amylase was then weighed to its working concentration as described previously (500 U/mL for AO; 10% *w/v* for BS), and then added to the diluted antibiotic solutions. Next, 1:2 serial dilutions were completed in a 96-well plate using the prepared solutions in a corresponding α-amylase + LB solution to ensure the α-amylase was not diluted. The resulting antibiotic concentration ranges were 1000–2 μg/mL and 100–0.2 μg/mL. Following serial dilution, the wells were inoculated with 20 μL of PAO1 overnight culture adjusted to OD_600_ 0.5. A column containing α-amylase + LB with no inoculant served as a negative control, and another containing α-amylase + LB with 20 μL inoculant served as a positive control. Inoculated 96-well plates were incubated for 24 h at 37 °C and 220 rpm shaking. After incubation, a plate-reading spectrophotometer (SpectraMax^®^, Molecular Devices) recorded absorbance at 600 nm wavelength to measure cell growth. MICs with α-amylase were defined as the lowest concentration in which the absorbance was similar to that of the α-amylase + LB-only negative control. Comparisons between the MICs for each antibiotic alone against the antibiotics with either homolog of α-amylase were made. Increased MIC values in the presence of α-amylase indicated potential antibiotic interaction, while equal MIC values indicated no observable antibiotic interaction.

### 4.7. Determination of Multiple Reaction Monitoring (MRM) parameters 

LC-MS/MS was conducted on a Shimadzu triple quadrupole LCMS-8045 with UHPLC system with the autosampler in electrospray ionization mode. A Nexcol C18 reverse phase column (2.1 × 50 mm, 1.8 µm, Part Number: 220-91394-08) using a flow rate of 0.8 mL/min and a gradient mobile phase of 10–97% acetonitrile in 0.1% aqueous formic acid over 5 min. Antibiotics (1 µL, 0.1 mg/mL) were injected into the LC-MS/MS instrument. MRM transitions were monitored in the positive ionization mode. MRM and retention times for each antibiotic are as follows: tetracycline 445.1/426.2 *m*/*z*, 2.017 min; ciprofloxacin 332.3/314.1 *m*/*z*, 1.995 min; levofloxacin 362.4/317.8 *m*/*z*, 1.942 min; colistin 578.61/101.2 *m*/*z*, 1.892 min; gentamicin 478.4/157.2 *m*/*z*, 0.103 min; tobramycin 468.4/163.2 *m*/*z*, 0.101 min.

### 4.8. Stability Assays

1 µM tetracycline, ciprofloxacin, or levofloxacin was incubated with 1% α-amylase AO or BS for 22 h in 0.85% NaCl at 37 °C in a final volume of 200 µL. Colistin and the aminoglycosides were evaluated at higher concentrations due to the lower limits of detection by LC-MS/MS. Briefly, 5 µM colistin, 7 µM gentamicin, or 7 µM tobramycin were incubated with 1% α-amylase AO or BS for 22 h in 0.85% NaCl at 37 °C in a final volume of 200 µL. Three technical replicates for each antibiotic and each α-amylase were conducted. A sample of antibiotic without α-amylase served as a negative control (3 technical replicates). After incubation, proteins were precipitated with 400 µL acetonitrile, vortexed, and centrifuged at 13,000 rpm for 15 min to precipitate protein. The supernatant was transferred to a clean microcentrifuge tube and concentrated to dryness using a ThermoScientific Savant SC250EXP SpeedVac concentrator (Waltham, MA, USA) for 2 h at 45 °C. The resulting dry antibiotic residue was suspended in 167 µL deionized water. Due to these processing steps, the actual concentration of each antibiotic, assuming no degradation, is as follows: 1.19 µM tetracycline, 1.19 µM ciprofloxacin, 1.19 µM levofloxacin, 5.99 µM colistin, 8.38 μM gentamicin, and 8.38 μM tobramycin. Then, 5 μL of each sample or standards of known concentration (0–10 µM) was injected into the LC-MS/MS and monitored using the appropriate MRM transitions. Antibiotic concentration in each sample was calculated by interpolation from a standard curve.

### 4.9. Differential Scanning Fluorimetry Assays

Differential scanning fluorimetry assays were performed using a BioRad CFX96 qPCR instrument (Hercules, CA, USA) in the FRET channel. Each antibiotic was screened under the following conditions: 1.0 mg/mL α-amylase, 1 mM antibiotic, and 6.25 × SYPRO Orange (ThermoScientific Waltham, MA, USA; CAT# 89833) in 0.85% sodium chloride. Controls containing protein only (1.0 mg/mL α-amylase, 0 mM antibiotic, and 6.25 × SYPRO), antibiotic only (1 mM antibiotic and 6.25 × SYPRO), no protein/no antibiotic (6.25 × SYPRO), or lysozyme positive control (500 µg/mL, 6.25 × SYPRO) in 0.85% sodium chloride were also included. Assays were conducted with four replicates per antibiotic or control at a final volume of 20 µL in optically clear qPCR strip tubes (USA Scientific Ocala, FL, USA; CAT#1402-2500). Briefly, strip tubes were placed in an ice bath before the sequential addition of 0.85% sodium chloride, protein, antibiotics or vehicle, and SYPRO. Melting temperatures were determined using DSFWorld San Francisco, CA, USA [68].

### 4.10. Data Analysis

Statistical analyses were performed using GraphPad Prism (GraphPad Software v10.1.1, LLC. Boston, MA, USA). Analyses performed include one- and two-way analysis of variance (ANOVA), followed by Tukey’s multiple comparison test.

## 5. Conclusions

This study demonstrates that the process of determining optimal α-amylase + antibiotic anti-biofilm treatments is complex, requiring the consideration of many different factors that can impact treatment efficacy. These include the choice of antibiotic, organismal source of α-amylase, and various experimental conditions such as treatment exposure length.

As this study only sought to propose and document the presence of counter-productive α-amylase + antibiotic interactions, more research is needed to better understand the mechanisms through which the previously discussed phenomena occur. Firstly, the chemical mechanisms underlying the degradation and sequestration of various antibiotics by α-amylase should be determined. It is possible that enzyme degradation or sequestration is occurring, it may require higher concentrations of enzyme to see this effect. Additionally, an array of homologs of α-amylase should be screened for *P. aeruginosa* biofilm dispersion efficacy and antibiotic compatibility to determine the best organismal source of α-amylase for therapeutic applications. LC-MS/MS stability and binding assays should be continued as a way of directly assessing the compatibility of combinatorial α-amylase + antibiotic anti-biofilm treatments, supported by in vitro and in vivo assessment of these treatments. It is important to also note that there are natural enzymes present within a biofilm produced by the bacteria that may also have the ability to degrade the antibiotic, GH, or both. Future work could focus on treatment degradation via bacterial macromolecules and possible EPS sequestration. These gaps in knowledge should be swiftly addressed, as they will greatly streamline the process of choosing the most optimal α-amylase + antibiotic combinations for therapeutic use.

## Figures and Tables

**Table 1:** MIC Values Change Upon Exposure to α-Amylase AO and BS. *n* = 2–29.

Antibiotic	Original MIC (µg/mL)	α-Amylase AO (µg/mL)	α-Amylase BS (µg/mL)
Ciprofloxacin	0.4	0.78	1.563
Levofloxacin	1.5	3.125	6.25
Colistin	31.25	31.25	15.63
Tobramycin	6.25	6.25	25
Gentamicin	3.125	3.125	25
Tetracycline	31.25	>1000	500

## Data Availability

Please contact the corresponding author for original data sets.

## Supplementary Materials

The following supporting information can be downloaded at https://www.mdpi.com/article/10.3390/antibiotics14090941/s1, Figure S1: MIC s in the presence or absence of α-amylase AO or BS; Table S1: Antibiotic MIC and data sheet; Figure S2: Effects of buffers on α-amylase melting temperatures.
