# Supplementary material for: α-Amylase-Mediated Antibiotic Degradation and Sequestration in Pseudomonas aeruginosa Biofilm Therapy
Source: Antibiotics (Basel). 2025 Sep 18;14(9):941. doi: 10.3390/antibiotics14090941 (PMC12466716; doi:10.3390/antibiotics14090941)
Supplement: Supplementary file 1 [file antibiotics-14-00941-s001.zip › antibiotics-3799491-supplementary.pdf]

Supplemental Figures:

Supplemental Table 1. Antibiotic MIC and Data Sheet.

Antibiotic	MIC (µg/mL)	Class	Effect on Microbial Cells	Charge (pH 7)	Targeted Structures / Mechanisms
**Ciprofloxacin**	0.4	Fluoroquinolone	Bactericidal	Neutral	DNA synthesis
**Levofloxacin**	1.5	Fluoroquinolone	Bactericidal	Neutral	DNA synthesis
**Colistin sulfate**	31.25	Polymyxin	Bactericidal	Positive	Bacterial Membranes
**Tobramycin**	6.25	Aminoglycoside	Bactericidal	Positive	Protein Translation
**Gentamicin**	3.125	Aminoglycoside	Bactericidal	Positive	Protein Translation
**Tetracycline**	31.25	Tetracycline	Bacteriostatic	Negative/Neutral	Protein Translation

antibiotics

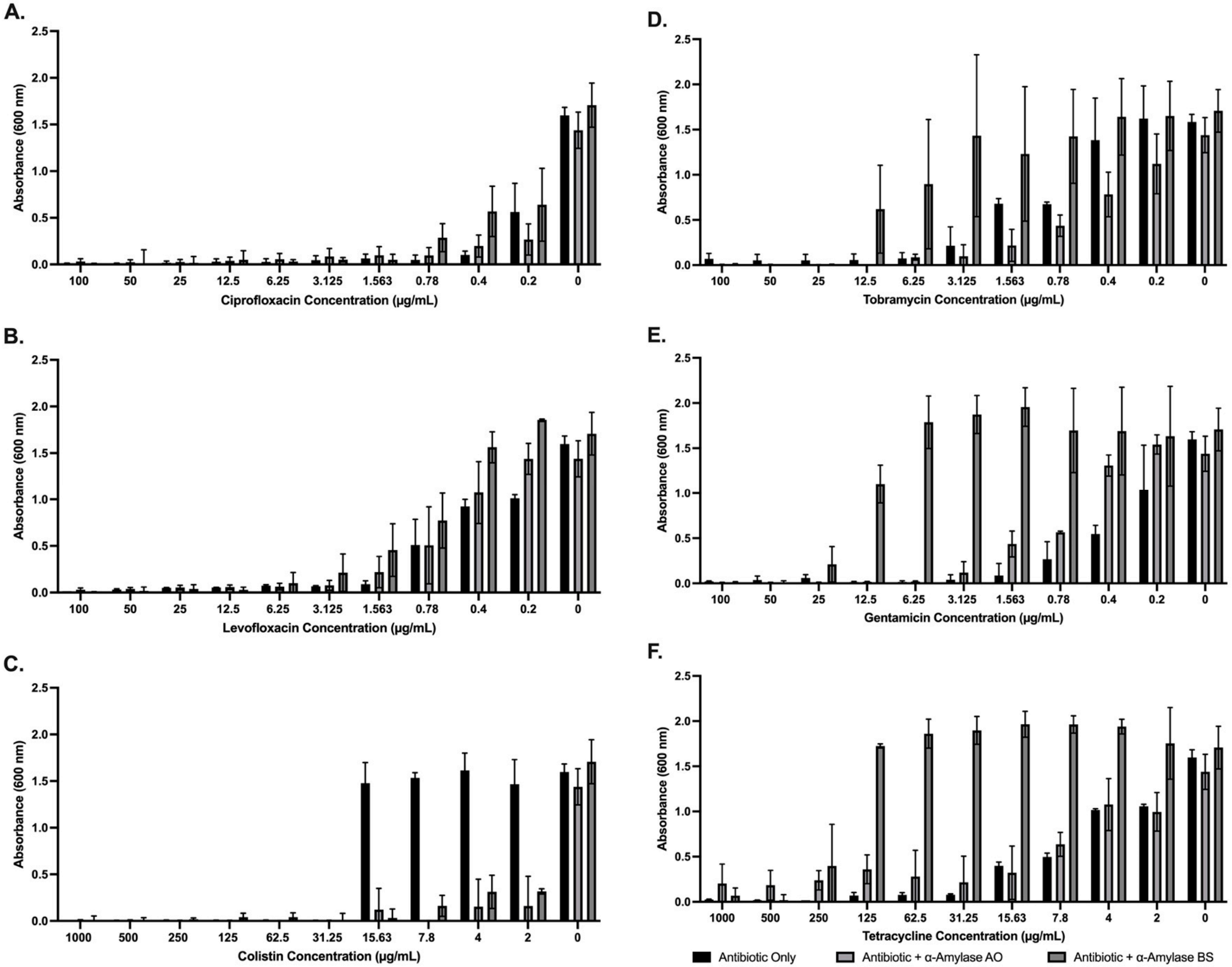

Supplemental Figure 1. MIC s in the Presence or Absence of α-amylase AO or BS. OD-adjusted cultures were inoculated into a 96-well plate with either media alone (control), antibiotic alone (black), antibiotic + α-amylase AO (light gray), or antibiotic + α-amylase BS (dark gray). The absorbance was measured at 600 nm and normalized to the absorbance of media alone. MICs were determined as the lowest concentration of antibiotic with 99% inhibition. . n = 2-29.

antibiotics

MDPI

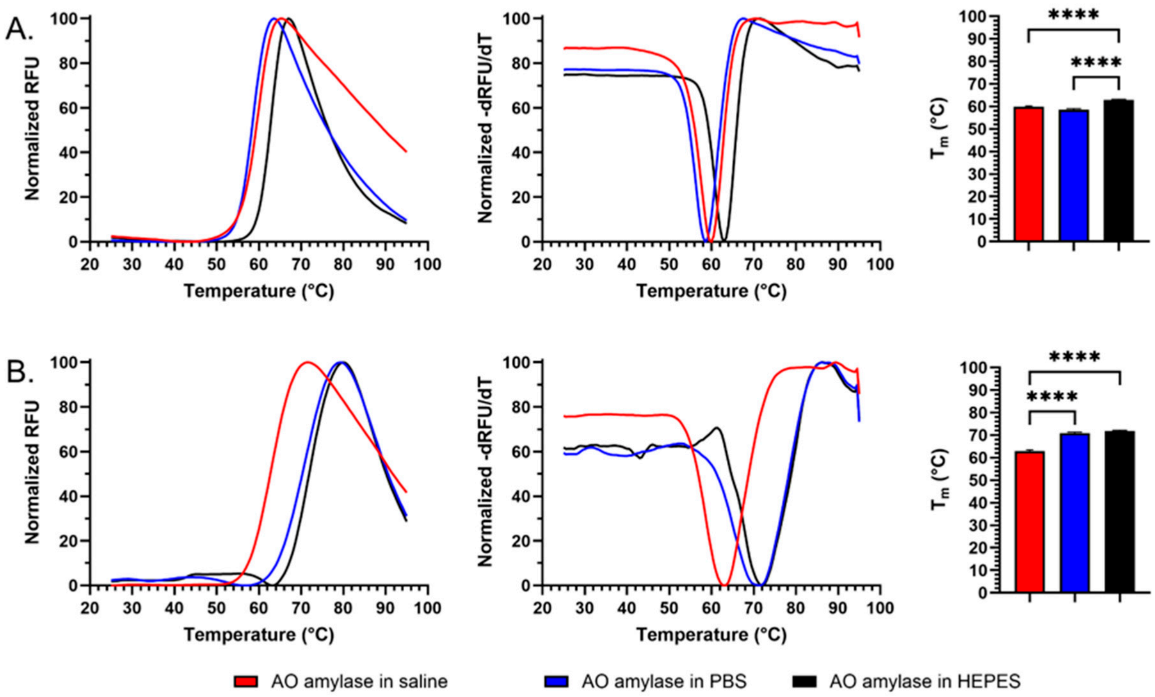

Supplemental Figure 2. Effect of buffers on amylase melting temperatures. Normalized fluorescence of SYPRO Orange (left), first derivative plots (center), and melting temperatures (right) for (A) 1.0 mg/mL α-amylase AO or (B) α-amylase BS in various buffers. Saline is 0.85% sodium chloride, PBS = phosphate-buffered saline, HEPES = 2-[4-(2-hydroxyethyl)piperazin-1-yl]ethanesulfonic acid. Statistical significance was determined via one-way ANOVA and Tukey's multiple comparison test. *, $p < 0.05$, **, $p < 0.01$, ***, $p < 0.001$, ****, $p < 0.0001$, n =4.
